# The *FoPLT* Gene of *Fusarium oxysporum* Affects Conidial Development and Pathogenicity

**DOI:** 10.3390/jof12030194

**Published:** 2026-03-09

**Authors:** Xiaoqi Han, Yanglin Zhang, Tianhao Fu, Yinuo Liu, Yanzhao Zhu, Yanan Wang, Xianglong Meng, Pengbo Dai, Keqiang Cao, Bo Li, Shutong Wang

**Affiliations:** 1 College of Plant Protection, Hebei Agricultural University, Baoding 071001, China; 15230398331@163.com (X.H.); 15128968662@163.com (Y.Z.); a44748218@126.com (T.F.); 18631961670@163.com (Y.L.); wyn3215347@163.com (Y.W.); cugmxl@163.com (X.M.); daipengbo@hebau.edu.cn (P.D.); cao_keqiang@163.com (K.C.); 2 Xingtai Academy of Agricultural Sciences, Xingtai 054000, China; 15731966706@163.com

**Keywords:** apple replant disease, *Fusarium oxysporum*, pathogenicity

## Abstract

Apple replant disease (ARD) is a soil-borne disease that severely restricts root development in orchards, impedes tree growth, and leads to reduced yields and decreased fruit quality, and thus significant economic losses. Previous studies identified *Fusarium oxysporum* as a major pathogenic agent. In this study, a T-DNA insertion mutant library of 13,000 *F. oxysporum* HS2 strains was utilized to screen for mutants with impaired pathogenicity. Nine mutants exhibiting reduced virulence were obtained, and the insertion sites of five mutants were successfully identified. Among them, we selected the HS2-29 strain, which exhibited the most significant decrease in conidial production, for further investigation. Its T-DNA was inserted into the *FoPLT* gene. RT-qPCR analysis revealed that the expression of the *FoPLT* gene rapidly increased during the early infection stage, followed by a decline and eventual stabilization. After the deletion of the *FoPLT* gene, the production of aerial hyphae, conidial yield, conidial length, and conidial diameter all significantly decreased. Stress tolerance assays indicated that *FoPLT* does not affect cell wall integrity in *F. oxysporum*. The deletion of the *FoPLT* gene significantly reduced the pathogenicity of *F. oxysporum*, and inoculating *Malus robusta* seedlings with the *FoPLT* knockout mutant led to significant increases in plant height, root length, fresh weight, and dry weight. These results suggest that the *FoPLT* gene plays a critical role in the pathogenicity of *F. oxysporum*.

## 1. Introduction

Apple replant disease (ARD), alternatively termed apple continuous cropping disorder or apple replant problem, constitutes a significant phytopathological challenge affecting major apple-producing regions worldwide. This complex disease syndrome manifests through severe root system inhibition, stunted tree growth, and substantial reductions in both fruit yield and quality, resulting in considerable economic losses for apple growers [1]. The etiology of ARD involves a multifaceted interplay of biotic and abiotic factors [2,3], with soilborne pathogens from multiple microbial groups identified as primary causal agents. Extensive research has implicated fungal genera including *Fusarium*, *Cylindrocarpon*, *Rhizoctonia*, *Phytophthora*, and *Pythium* as key contributors to ARD pathogenesis [3,4], though their relative importance may vary across different orchard systems and geographical regions.

Among these pathogenic organisms, *Fusarium oxysporum* has emerged as particularly noteworthy due to its global distribution, extensive host range, and remarkable genetic adaptability. As a soilborne filamentous fungus, *F. oxysporum* demonstrates the capacity to infect over 100 economically important plant species, including cotton, corn, banana, various cereals, tomatoes, and peanuts, typically causing devastating Fusarium wilt diseases that are notoriously difficult to manage [5,6,7]. The pathogen’s remarkable host specificity, reflected in its classification into numerous formae speciales, each containing multiple physiological races, coupled with its genetic polymorphism and high variability, has established *F. oxysporum* as a focal organism in contemporary plant pathology research [8]. The recent availability of complete genome sequences for various physiological races and formae speciales has further elevated its status as a model system for investigating fundamental questions in developmental biology, microbiology, and molecular plant pathology, particularly in studies of pathogen–host interactions.

The pathogenic success of *F. oxysporum* is derived from its sophisticated arsenal of virulence mechanisms, which operate through several coordinated systems. First, the fungus produces an extensive array of virulence factors that facilitate host colonization, including various cell wall-degrading enzymes (CWDEs) that systematically break down plant cell wall components [9], as well as toxic secondary metabolites like fusaric acid that induce cellular damage [10]. Second, *F. oxysporum* employs complex signal transduction networks involving MAPK cascades, Ras-mediated signaling, G-protein-coupled pathways, velvet family proteins (LaeA/VeA/VelB), and cAMP-dependent pathways to precisely regulate virulence gene expression during infection [11]. Third, genetic studies using protoplast-mediated transformation and gene knockout techniques have identified specific genes, including *FTF*, *FgPEX1/10*, and *PTC6*, that play crucial roles in pathogenicity [12,13,14].

Notably, the functional importance of CWDEs in *F. oxysporum* pathogenesis has been demonstrated through multiple experimental approaches. While Snf1 gene knockout mutants exhibited significantly reduced virulence in model plants like *Arabidopsis thaliana* and cabbage [15], suggesting an important role for pectinases in pathogenesis, studies of individual CWDE genes have revealed context-dependent effects. For instance, the cotton-specific *Fovpg1* gene product significantly influences disease symptom development [16], while in tomato pathogens, single deletions of either endoPGs (*pg1*) or exoPGs (*pg5*) showed minimal effects on pathogenicity, though their combined deletion substantially reduced virulence [9]. Similarly, in *F. oxysporum* f. sp. *Cubense* race 4 (*Foc4*), disruption of exoPGs (*pgx4*) markedly decreased pathogenicity and impaired aerial hyphal growth [17]. These findings collectively demonstrate that CWDEs often function synergistically in *F. oxysporum* pathogenesis, with their relative contributions varying across different host–pathogen systems.

In this study, using the T-DNA insertion mutant library of *F. oxysporum* strain HS2, previously constructed by our research group, we screened for mutants with reduced pathogenicity, identified their T-DNA insertion sites, and cloned a pathogenicity-related gene, *FoPLT*. By studying the role of the *FoPLT* gene in the growth, development and infection of *F. oxysporum*, we investigated its pathogenic mechanisms and provided a theoretical basis for studying interactions between pathogenic fungi and their host plants.

## 2. Materials and Methods

### 2.1. Tested Strains and Plasmids

The *F. oxysporum* HS2 strain and the *F. oxysporum* T-DNA insertion mutant library were provided by our laboratory (Plant Disease Epidemiology and Integrated Prevention and Control Laboratory, College of Plant Protection, Hebei Agricultural University) and stored in glycerol at −80 °C. *F. oxysporum* HS2 was isolated from ARD in our laboratory. The plasmid pUChyg, containing the hygromycin resistance gene *hyg*, and the plasmid pGTN, containing the G418 resistance gene, were also provided by our laboratory.

### 2.2. Screening of Virulence-Weakening Mutants of F. oxysporum

Fresh mung beans were placed in a Petri dish lined with water-soaked gauze and incubated at a constant temperature of 20 °C for 24 h to allow germination. Germinated mung beans with similar radicle lengths were selected using sterilized tweezers, soaked in 75% ethanol for 30 s, and then transferred to 1% sodium hypochlorite for another 30 s. Afterward, the beans were rinsed three times in distilled water on a shaker at 37 °C and 200 rpm for 5 min each. Surface moisture was removed using sterilized filter paper, and the beans were placed on water agar plates with their radicles oriented toward the center of the colony (five beans per dish). The plates were incubated at 25 °C for 5 d to observe disease susceptibility, and radicle lengths were measured to compare the pathogenicity of the mutants. Three biological replicates employed per mutant in the mung bean assay.

The *F. oxysporum* HS2 strain, the deletion mutant (Δ*FoPLT*) of the *FoPLT* gene and the complementation mutant (Δ*FoPLT*-C) of the *FoPLT* gene were cultured on a PDA plate at 25 °C for 6 days. A 5 mm diameter fungal plug was extracted from each active mutant using a sterile punch and inoculated onto a fresh PDA plate with the mycelium surface facing downward. The plates were incubated upside-down at 25 °C for 6 d, after which the colony diameter of each mutant was measured. Colony diameter was measured along two perpendicular axes.

For conidial concentration analysis, Δ*FoPLT*, Δ*FoPLT*-C and the wild-type HS2 strain were cultured in a 25 °C incubator for 6 d. A 5 mm diameter fungal plug was transferred to a conical flask containing 50 mL of YPD liquid medium (one cake per flask) and cultured on a shaker at 25 °C and 180 rpm for 3 d. The filtrate was collected using sterilized filter cloth, and conidial concentration was quantified using a hemocytometer.

### 2.3. Bioinformatics Analysis

Based on the HS2 genome database from our laboratory, the software TBtools-II v2.390 was used for comparative analysis to determine the genomic location of the *FoPLT* gene and its nucleotide and amino acid sequences. The conserved domains of the *FoPLT* gene were predicted and analyzed using the CD-Search tool on NCBI (30 May 2023). Subcellular localization, signal peptides, transmembrane domains, and physicochemical properties of the *FoPLT* protein were predicted using CELLO2GO v2.0, SignalP v4.1 Server, TMHMM Server v.2.0, and ProtParam tool.

### 2.4. Construction of Knockout and Complementation Strains

To construct the *FoPLT* deletion mutant, genomic DNA was first extracted from the wild-type strain using the CTAB method. The upstream and downstream fragments of the *FoPLT* gene were amplified from the HS2 genomic DNA by PCR. Simultaneously, the hygromycin resistance gene *hyg* was amplified from the pUC-hyg plasmid DNA using hF-hR primers. The *FoPLT* gene fragments and the *hyg* gene were seamlessly cloned into the pkNTG vector using the All-in-One Gold Seamless Cloning Kit.

For the *FoPLT* complementation mutant, *F. oxysporum* DNA was used as a template. A *BamH*I site was introduced at the 5′ end and a *Cla*I site at the 3′ end of the complementation fragment, matching the restriction sites of the pGTN vector. The fragment and vector were digested with the respective restriction enzymes and ligated using T4 DNA ligase.

### 2.5. Observation of Biological Phenotypes of F. oxysporum

The wild-type HS2 strain and its knockout and complementation mutants were cultured on PDA solid medium plates. Colony morphology and growth were observed and recorded after incubation at 25 °C for 6 d.

Sterilized cover glasses were evenly placed on solidified PDA plates. Activated wild-type HS2 and mutant strains were inoculated onto the plates and cultured at 25 °C for 3–4 d. The cover glasses with adhered hyphae were removed using sterilized tweezers, and mycelial morphology was observed under a microscope.

For conidial production analysis, activated wild-type HS2 and mutant strains were inoculated into sterilized Potato glucose liquid culture medium (three 5 mm diameter fungal plugs per flask) and cultured on a shaker at 25 °C and 175 rpm for 3 d. Mycelia were removed by filtration, and the conidial concentration was quantified using a hemocytometer (five-point method). Conidia were diluted to 1 × 10^6^ conidia/mL, and 100 µL of the suspension was pipetted onto water agar plates. After incubation at 25 °C for 6–8 h, conidial germination was observed under a microscope.

### 2.6. Mung Bean Inoculation and Infection

Fresh mung beans (*Vigna radiata*) were germinated as described above. Uniformly germinated beans were surface-sterilized and soaked in mutant conidial suspensions, then shaken at 37 °C and 200 rpm for 30 min to ensure thorough contact. The beans were dried on absorbent paper and placed on water agar (WA) plates containing ampicillin. After incubation at 28 °C for 24 h, disease susceptibility was observed, and radicle lengths were measured to compare pathogenicity.

### 2.7. Potted M. robusta Seedlings

*F. oxysporum* was cultured on PDA plates at 25 °C for 6 d. Five-millimeter-diameter fungal plugs were inoculated into sterilized cooked wheat in wide-mouth bottles and incubated at 25 °C for 15 d, with periodic addition of sterile water. The inoculum was mixed with nutrient soil and vermiculite (1:5:5 ratio), and three-leaf-stage *M. robusta* seedlings were planted in the mixture. Pathogenicity was assessed after four weeks of greenhouse cultivation.

## 3. Results

### 3.1. Screening and Identification of the FoPLT Gene in F. oxysporum

**Figure 1:**
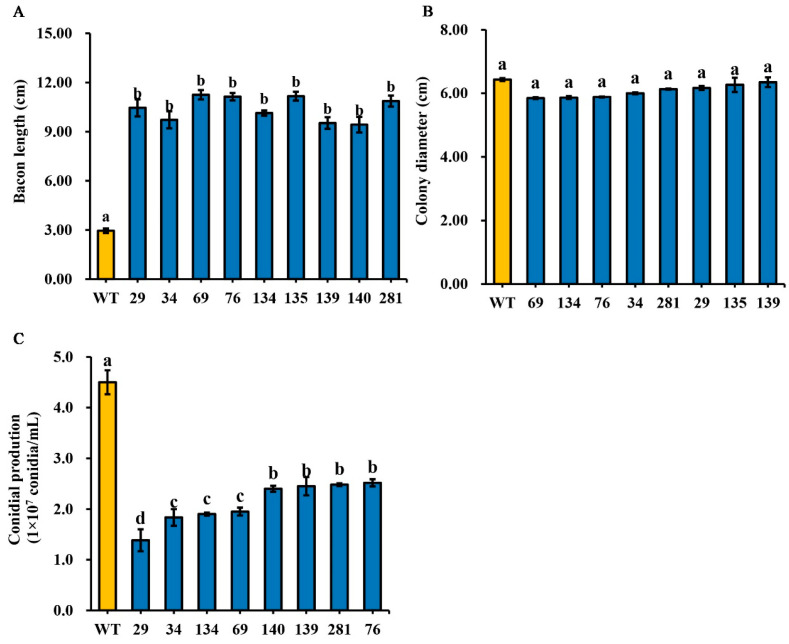
Pathogenicity assay (**A**), and determination of colony growth rate (**B**) and conidial production (**C**) of nine *F. oxysporum* mutants. The abscissa axis is the strain name. The mean values ± SD were calculated from three independent experiments. For multiple comparisons, different lowercase letters (a, b, c) denote significant differences at *p* < 0.05. Three biological replicates were employed per treatment.

To identify *F. oxysporum* mutants with reduced pathogenicity, we screened 13,000 T-DNA insertion mutants derived from the wild-type HS2 strain. Nine mutants (HS2-29, HS2-34, HS2-69, HS2-76, HS2-134, HS2-135, HS2-139, HS2-140, and HS2-281) exhibited significantly attenuated virulence (Table S1). Mung beans inoculated with these mutants showed radicle lengths 3.54-, 3.29-, 3.81-, 3.77-, 3.44-, 3.79-, 3.23-, 3.20-, and 3.68-fold longer, respectively, than those infected by the wild-type HS2 strain (Figure 1A).

The colony growth test showed no significant difference between the mutant and HS2 (Figure 1B). However, compared to HS2, the conidial production in the mutants significantly decreased by 69.26% (HS2-29), 59.26% (HS2-34), 57.78% (HS2-69), 56.67% (HS2-76), 46.67% (HS2-134), 45.56% (HS2-135), 44.89% (HS2-139), 44.07% (HS2-140), and 42.59% (HS2-281) (Figure 1C).

Flanking sequences of T-DNA insertion sites were amplified and aligned with the HS2 genome. Five mutants (HS2-29, HS2-69, HS2-134, HS2-139, HS2-140) were mapped to specific genes: transporter protein (HS2-29), protein kinase (HS2-69), transferase (HS2-134), keratinase (HS2-139), and reductase (HS2-140) (Table S2). The HS2-29 mutant, with the most severe conidial production defect, harbored a disruption in the *FoPLT* gene. Bioinformatic analysis indicated that *FoPLT* encodes a 1304-amino-acid protein with five transmembrane domains and a conserved HAD-like (haloacid dehalogenase) domain (Figure S2). The HAD superfamily includes P-type ATPases, confirming *FoPLT* as a putative P-type ATPase.

### 3.2. FoPLT Is Involved in F. oxysporum Vegetative Growth

**Figure 2:**
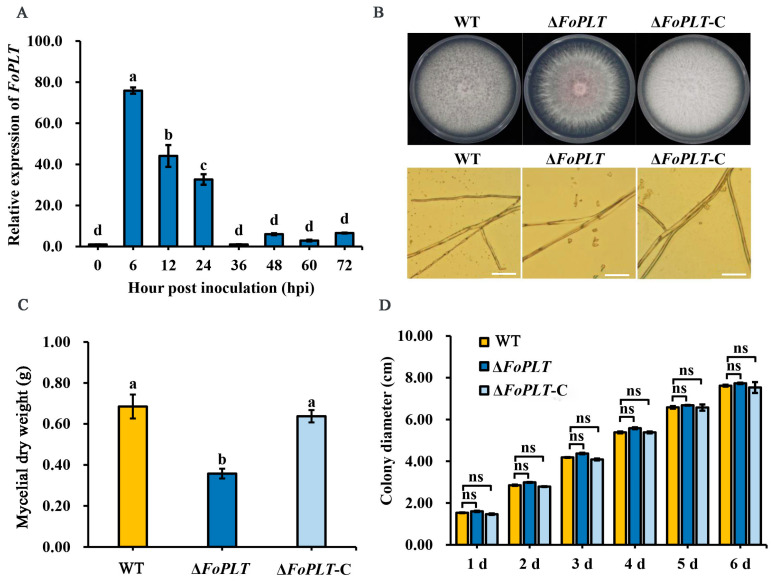
Analysis of *FoPLT* expression patterns and growth phenotype analysis of *FoPLT* deletion mutants, complementation strains, and WT. (**A**) The expression levels of *FoPLT* in the WT strain at different time points (0 hpi, 6 hpi, 12 hpi, 24 hpi, 36 hpi, 48 hpi, 60 hpi, 72 hpi) after inoculation were compared with those during the mycelial stage. The mean values ± SD were calculated from three independent experiments. For multiple comparisons, different lowercase letters (a, b, c, d) denote significant differences at *p* < 0.05. *Actin* gene of *F. oxysporum* was used as an internal reference gene. Six biological replicates were employed per treatment. (**B**) Mycelial morphology and microscopic observation of hyphae of the WT, Δ*FoPLT*, and Δ*FoPLT*-C strains. Bar, 25 µm. Three biological replicates were employed per treatment. (**C**) Mycelial dry weight of the WT, Δ*FoPLT*, and Δ*FoPLT*-C strains grown on PDA plates for 6 d. The mean values ± SD were calculated from three independent experiments. For multiple comparisons, different lowercase letters (a, b) denote significant differences at *p* < 0.05. Four biological replicates were employed per treatment. (**D**) Colony diameter of the WT, Δ*FoPLT*, and Δ*FoPLT*-C strains. The abscissa axis is different days. The mean values ± SD were calculated from three independent experiments. Two-tailed Student’s *t*-tests were conducted; ns means not significant. Six biological replicates were employed per treatment.

RT-qPCR analysis revealed that *FoPLT* expression peaked 6 h post-infection (hpi) before declining to baseline, suggesting its involvement in early infection stages (Figure 2A). We constructed the deletion mutant Δ*FoPLT* and the complementation mutant Δ*FoPLT*-C of the *FoPLT* gene (Figure S1). We examined the growth of Δ*FoPLT*, and of complementation strains cultured on PDA media plates for 6 d. The mycelia of Δ*FoPLT* mutants did not differ morphologically from those of the WT and complementation strains (Figure 2B). In addition, Complementation restored aerial hyphae to wild-type levels. The dry weight of mycelium in Δ*FoPLT* was significantly reduced compared to HS2 and Δ*FoPLT*-C (Figure 2C). There was no difference in colony growth rate between Δ*FoPLT*, Δ*FoPLT*-C, and HS2 (Figure 2D). These results indicate that *FoPLT* does not affect colony growth but affects mycelium production.

### 3.3. FoPLT Affects Conidial Formation of F. oxysporum

**Figure 3:**
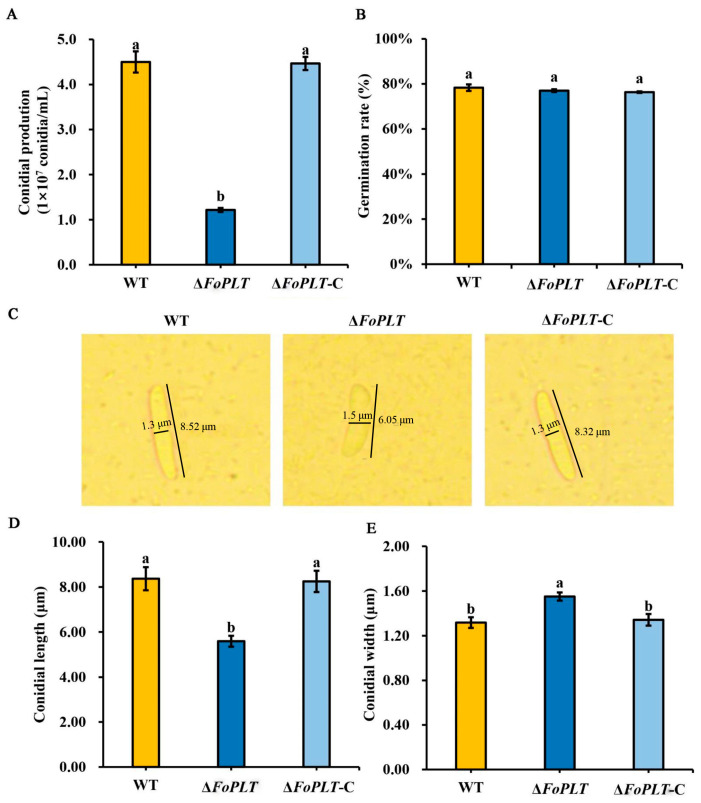
Conidial development and maturation analysis of the *FoPLT* deletion mutants, complementation strains, and WT strains. (**A**) Conidial production of the WT, Δ*FoPLT*, and Δ*FoPLT*-C strains. (**B**) Germination rate of the WT, Δ*FoPLT*, and Δ*FoPLT*-C strains. Three biological replicates were employed per treatment. (**C**) Microscopic (400×) observation of conidia morphology of the WT, Δ*FoPLT*, and Δ*FoPLT*-C strains. (**D**) Conidial length of the WT, Δ*FoPLT*, and Δ*FoPLT*-C strains. (**E**) Conidial width of the WT, Δ*FoPLT*, and Δ*FoPLT*-C strains. The mean values ± SD were calculated from three independent experiments. For multiple comparisons, different lowercase letters (a, b) denote significant differences at *p* < 0.05. Six biological replicates were employed per treatment.

After culturing in PDB medium for 3 d, we measured the conidial yield of Δ*FoPLT* and its complementary strain. We also calculated their conidial germination rates. Subsequently, we observed the morphology of their conidia. However, Δ*FoPLT* exhibited a 72.96% reduction in conidial yield (Figure 3A), though conidial germination rates were unaffected (Figure 3B). Conidia of Δ*FoPLT* were shorter but wider than those of HS2 (Figure 3C–E). The above results indicate that *FoPLT* affects the conidial production and conidial morphology of HS2.

### 3.4. FoPLT Does Not Affect Cell Wall Integrity

**Figure 4:**
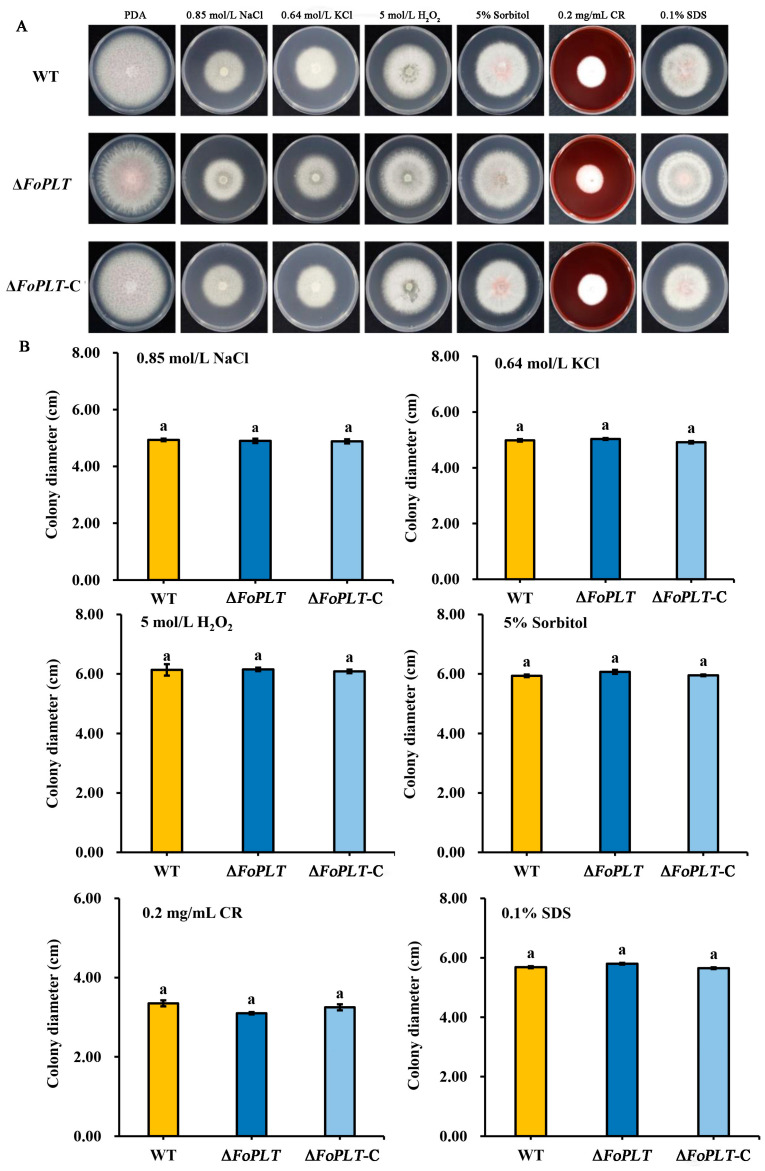
Stress resistance response (**A**) and histogram (**B**) of *F. oxysporum* mutants with *FoPLT* gene deletion and complementation. The abscissa axis is the name of different strains. The mean values ± SD were calculated from three independent experiments. For multiple comparisons, different lowercase letters (a) denote significant differences at *p* < 0.05. Six biological replicates were employed per treatment.

We conducted cell wall stress responses on strains Δ*FoPLT* and Δ*FoPLT*-C (0.85 mol/L NaCl, 0.64 mol/L KCl, 5 mol/L H_2_O_2_, 5% sorbitol, 0.2 mg/mL CR, 0.01% SDS) and found no significant difference between Δ*FoPLT* and WT and Δ*FoPLT*-C (Figure 4A). This demonstrates that knockout mutant strains are insensitive to abiotic stress in vitro. The above results indicate that *FoPLT* does not affect the integrity of the cell wall of *F. oxysporum* HS2 (Figure 4B).

### 3.5. FoPLT Affects the Pathogenicity of F. oxysporum Strain HS2

**Figure 5:**
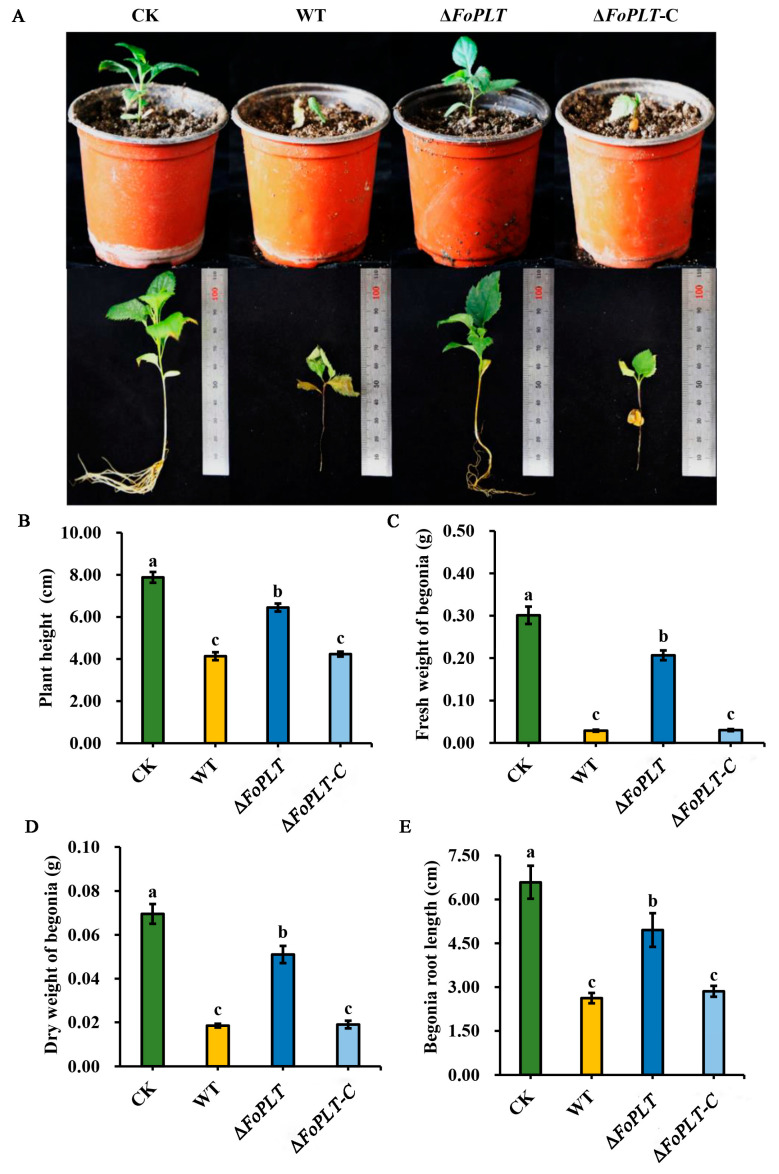
Variation in virulence for the *FoPLT* deletion mutants, complementation strains, and WT strain. (**A**) The growth status of *M. robusta* seedlings after 4 weeks of inoculation with CK (Blank control), WT, Δ*FoPLT*, and Δ*FoPLT*-C strains. Four weeks after inoculation, the seedlings were investigated and analyzed for growth indicators such as plant height (**B**), fresh weight (**C**), dry weight (**D**) and root length (**E**). Error bars indicate the standard deviation of the means. CK is a blank control with added water. The abscissa axis is the name of different strains. The mean values ± SD were calculated from three independent experiments. For multiple comparisons, different lowercase letters (a, b, c) denote significant differences at *p* < 0.05. Nine biological replicates were employed per treatment.

After removing *FoPLT* from HS2, its pathogenicity to *Malus robusta* was weakened (Figure 5A). The plant height of the gene deletion mutant Δ*FoPLT* (6.44 ± 0.57 cm) after inoculation was much higher than that of WT (4.13 ± 0.57 cm) and Δ -C (4.23 ± 0.35 cm) (Figure 5B), while the fresh weight of *M. robusta* after inoculation with WT (0.02 ± 0.01 g) and Δ*FoPLT*-C (0.03 ± 0.01 g) was much lower than that of Δ*FoPLT* (0.21 ± 0.03 g) (Figure 5C). Similarly, the dry weight of *M. robusta* after inoculation with WT (0.01 ± 0.01 g) and Δ*FoPLT*-C (0.01 ± 0.01 g) was much lower than that of Δ*FoPLT* (0.05 ± 0.01 g) (Figure 5D). After inoculation with Δ*FoPLT* (4.96 ± 1.73 cm), the root elongation of *M. robusta* was higher than that of WT (2.62 ± 0.53 cm) and Δ*FoPLT*-C (2.86 ± 0.56 cm) (Figure 5E). In summary, there are significant differences in growth indicators between the knockout mutant and the wild-type strain HS2. These results indicate that the deletion of the *FoPLT* gene leads to impaired pathogenicity of the HS2 strain.

## 4. Discussion

The wild-type strain HS2 used in this study is a highly pathogenic *F. oxysporum* isolate obtained from apple tree roots. The screening method for pathogenic mutants in fungal T-DNA insertion libraries varies depending on the fungal species and host plant characteristics. Routine pathogenicity testing using tree roots has disadvantages such as complex operation, time-consuming procedures, difficulty in observing symptoms, and significant differences between batches in the results. To overcome these limitations, we previously studied, established, and validated an embryo root inoculation experiment. Comparative analysis showed that the results of embryo root inoculation, the greenhouse pot experiment, and the field experiment were consistent [18]. The radicle inoculation method offers distinct advantages for pathogenicity assessment, including: easy access to experimental materials, simplified operational procedures, clear symptom observation and measurement, and high result reproducibility. Based on these validated advantages, we employed the radicle inoculation method for pathogenicity determination in this study.

In this study, we screened 300 mutants for pathogenicity from the previously established T-DNA insertion mutant library. Comparative analysis with the wild-type *F. oxysporum* HS2 strain showed that the pathogenicity of most mutants did not significantly change, with only a few mutants showing significant changes (Figure 1). The results are summarized as follows: Firstly, the randomness of T-DNA integration in fungal genomes may or may not affect genes associated with pathogenicity or phenotypic characteristics; secondly, there is a lack of consistent correlation between T-DNA insertion sites and observable phenotypic or pathogenic features; finally, the specific mutant strain detected, the transformation method used, and the copy number of the inserted T-DNA fragment are factors that collectively promote innovative methods for assessing the pathogenicity of *F. oxysporum*.

Previous studies have demonstrated that *Agrobacterium*-mediated transformation in fungi can yield both target fragment sequences and vector sequences [19]. Through local BLAST analysis comparing the hiTAIL-PCR amplified flanking sequences with our *F. oxysporum* HS2 genome database, we identified and subsequently eliminated four vector-derived sequences. This analysis ultimately confirmed the insertion sites in five mutant strains (HS2-29, HS2-69, HS2-134, HS2-139, HS2-140). Gene function analysis revealed that: HS2-29 encodes a transporter protein, HS2-69 encodes a protein kinase, HS2-134 encodes a transferase, HS2-139 encodes a keratinase, and HS2-140 encodes a reductase (Table S2). These identified insertion sites provide valuable genetic resources for elucidating phenotype-associated genes and pathogenic mechanisms in *F. oxysporum*.

Gene expression levels serve as direct indicators of transcriptional activity, reflecting the abundance of specific mRNA transcripts within cells [20]. Quantitative analysis of these expression patterns enables researchers to detect differential gene expression, examine gene–gene correlations, and evaluate functional changes under different physiological conditions.

To elucidate the relationship between *F. oxysporum* pathogenicity and *FoPLT* gene function, we monitored the expression dynamics of *FoPLT* at different infection stages via RT-qPCR. Our analysis revealed significantly elevated *FoPLT* expression during the early infection phase, suggesting its potential functional importance in initial host–pathogen interactions (Figure 2A).

In order to elucidate the functional role of *FoPLT* in the pathogenesis of *F. oxysporum*, this study generated *FoPLT* knockout and complementary mutants. The preliminary feature description focuses on comparative analysis of phenotypic characteristics, including colony morphology, hyphal development, spore morphology, sporulation ability, and spore germination efficiency between mutant and wild-type strains. The key observation results of knocking out the mutant include a significant reduction in aerial hyphal growth, a significant decrease in sporulation ability, and a significant decrease in spore size. These phenotypic changes indicate that *FoPLT* plays an important regulatory role in the growth and development of *F. oxysporum* (Figure 3).

Conidia is one of the important pathogenic factors of fungi. Previous studies have confirmed that Pde1p (a functional homolog of yeast Dnf3p) determines the virulence of *Magnaporthe grisea* by affecting spore function [21]. It is the same as the corresponding protein in yeast. LHS1 is seriously damaged not only in conidia formation, but also in nutrition and biological infection [22]. Fungal sporulation is closely related to amino acid biosynthesis. MoLYS2 is a key lysine biosynthesis gene, and its deletion leads to the defect of conidial production [23]. It is worth noting that this sporulation defect can be prevented by exogenous lysine supplementation, while restoring the pathogenicity of the knockout *strain Schizosaccharomyces pombe* encodes a spore-forming specific Pil1 family protein, Meu14, which plays a special role in the formation of anterior pore membranes during spore formation [24]. In addition, studies have shown that some genes can affect amino acid synthesis by controlling the synthesis of some enzymes, thereby affecting the formation of conidia. Proline dehydrogenase (PROH) is a key enzyme in the process of glutamate biosynthesis, and its deletion leads to a reduction in asexual spore production [25]. The deletion of the methylenetetrahydrofolate reductase (MTHFRs)-encoding gene MET12 resulted in a significant reduction in conidial production [26]. The deletion of LEU, the gene encoding 3-isopropyl malate dehydrogenase (3-IPMDH) required for leucine biosynthesis, led to the defect of conidia production [27]. However, whether FoPLT can affect sporulation by affecting amino acid production remains to be confirmed.

Apt1p, a member of the P4-ATPase family, can be used as a key regulator of *Cryptococcus neoformans* polysaccharide secretion and pathogenicity, and participate in fungal virulence regulation through fine-grained regulation of lipid transport across the bilayer [28]. P4-ATPase, as a lipid turnover enzyme, can actively mediate the transmembrane transport of phospholipids in the cell membrane, which is an important basis for core life activities such as vesicle formation and membrane transport; the four P4-ATPases encoded by the genome of *Cryptococcus neoformans* play a role in lipid turnover and the secretion pathway [29]. At the same time, the fungal plasma membrane H^+^-ATPase (pma1) maintains a transmembrane electrochemical gradient and membrane potential by pumping protons out of the cell, which plays an important role in cell physiological homeostasis [30]. However, the specific function of this P-type ATPase in the related pathways of this study has not been verified, and it still needs to be further explored.

## 5. Conclusions

In this study, a mutant HS2-29 with significantly reduced pathogenicity was screened by screening the mutant library. The insertion site of the mutant gene is the *FoPLT* gene, which belongs to the P-type ATPase family gene. The knockout of the *FoPLT* gene resulted in a reduction in aerial hypha, spore production, spore growth defects and pathogenicity, indicating that the *FoPLT* gene affected the aerial hypha, spore production, growth and development, and pathogenicity of *F. oxysporum*.

## Data Availability

The original contributions presented in this study are included in the article/Supplementary Materials. Further inquiries can be directed to the corresponding authors.

## Supplementary Materials

The following supporting information can be downloaded at: https://www.mdpi.com/article/10.3390/jof12030194/s1, Table S1. Selected mutant strains of *Fusarium oxysporum* with significantly reduced pathogenicity; Table S2. Localization of five mutant genes; Figure S1. Identification of *FoPLT* gene deletion and complementation mutants; Figure S2. Biological analysis of *FoPLT* gene.

## Abbreviations

*F. oxysporum*: *Fusarium oxysporum*; RT-qPCR: reverse transcription–quantitative polymerase chain reaction; DEGs: differentially expressed genes.
