# Supplementary material for: The FoPLT Gene of Fusarium oxysporum Affects Conidial Development and Pathogenicity
Source: J Fungi (Basel). 2026 Mar 9;12(3):194. doi: 10.3390/jof12030194 (PMC13028058; doi:10.3390/jof12030194)
Supplement: Supplementary file 1 [file jof-12-00194-s001.zip › Supplementary Figure S1,S2.pdf]

Supplementary Figure

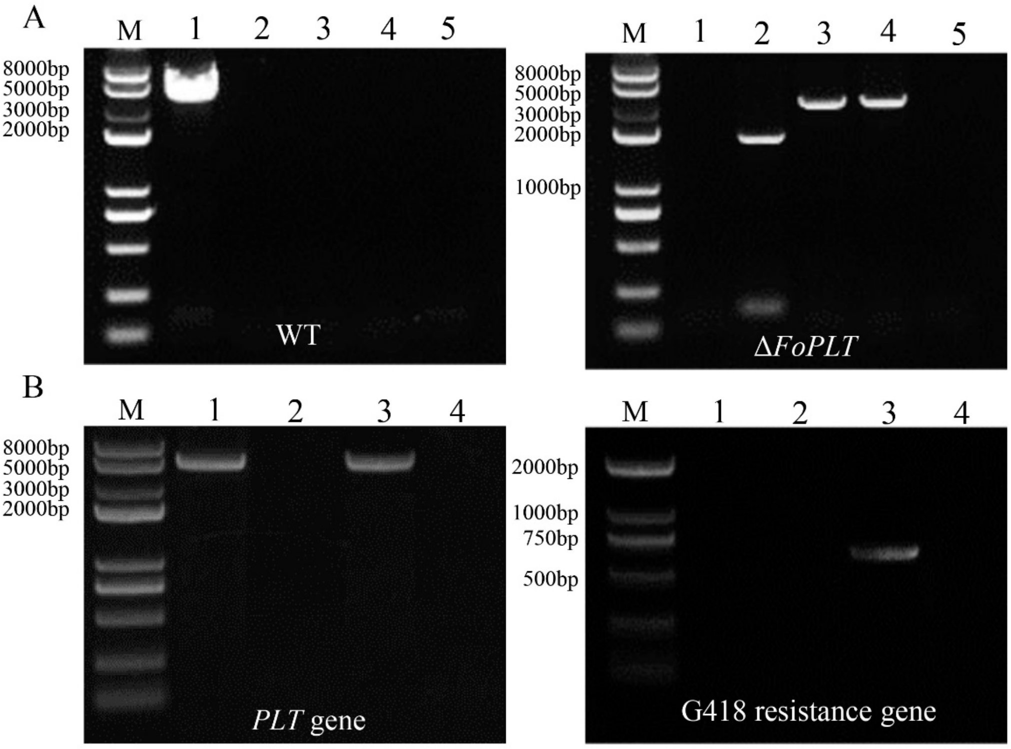

Figure S1 Identification of *FoPLT* gene deletion and complementation mutants (**A**) Identification of *FoPLT* gene deletion mutants. M: maker 1: detection of target gene *FoPLT*, 2: detection of hygromycin, 3: detection of upstream fragments, 4: detection of downstream fragments, 5: ddH_2O. (**B**) identification of *FoPLT* complementation mutants. M: Maker, 1: HS2, 2: Δ*FoPLT*, 3: Δ*FoPLT*-C, 4: ddH_2O.

A

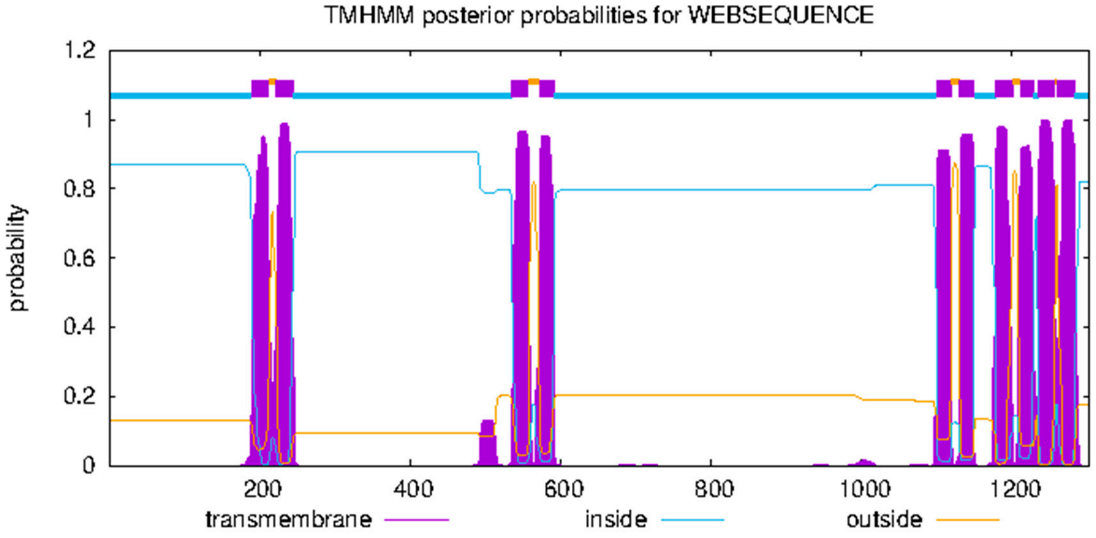

B

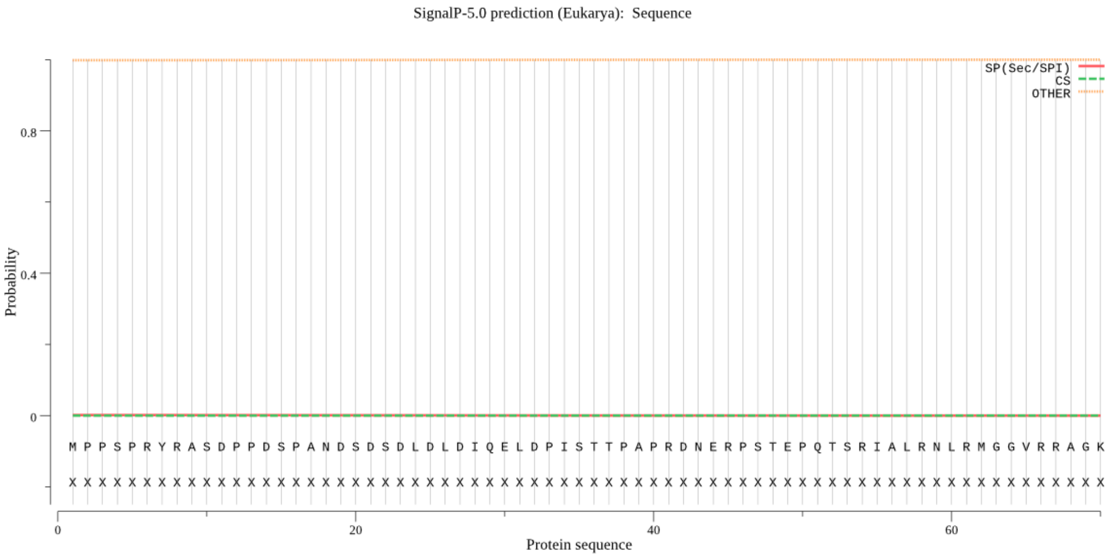

C

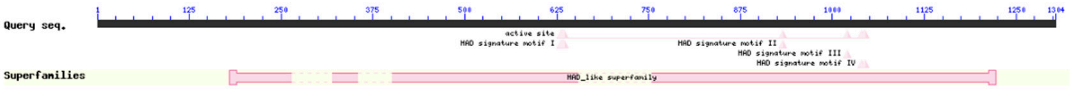

Figure S2. Biological analysis of *FoPLT* gene. **A** The gene contains a transmembrane domain. **B** The gene has no signal peptide. **C** The gene conserved domain.
